# Supplementary material for: Biodiversity conservation in cities: Defining habitat analogues for plant species of conservation interest
Source: PLoS One. 2020 Jun 9;15(6):e0220355. doi: 10.1371/journal.pone.0220355 (PMC7282666; doi:10.1371/journal.pone.0220355)
Supplement: S2 Material — (DOCX) [file pone.0220355.s005.docx]

Supplementary Material

**Table 2. Plant species co-occurring with *Matthiola crassifolia* in Beirut (* non-native species).**

| *Aegilops geniculata* Roth | *Erigeron bonariensis* L.* | *Phleum subulatum* (Savi) Asch. & Graebn. |
| --- | --- | --- |
| *Agave americana* L.* | *Erigeron canadensis* L.* | *Phyla nodiflora* (L.) Greene* |
| *Agave attenuata* Salm-Dyck* | *Euphorbia terracina* L. | *Picris rhagadioloides* (L.) Desf. |
| *Alcea setosa* (Boiss.) Alef. | *Ficus carica* L. | *Piptatherum miliaceum* (L.) Coss. |
| *Alyssum strigosum* Banks & Sol. | *Ficus microcarpa* L.f.* | *Pittosporum tobira* (Thunb.) W. T. Aiton* |
| *Amaranthus hybridus* L.* | *Galium canum* DC. | *Plantago coronopus* L. |
| *Ambrosia maritima* L. | *Galium murale* (L.) All. | *Plantago lagopus* L. |
| Anacamptis sancta (L.) R. M. Bateman | *Glebionis coronaria* (L.) Spach | *Polycarpon tetraphyllum* (L.) L. |
| *Anagallis arvensis* L. | *Hedypnois rhagadioloides* (L.) F. W. Schmidt | *Polygonum equisetiforme* Sm. |
| *Anchusa hybrida* Ten. | *Helichrysum stoechas* (L.) Moench | *Ricinus communis* L.* |
| *Anisantha rigida* (Roth) Hyl. | *Heliotropium hirsutissimum* Grauer | *Rostraria smyrnacea* (Trin.) H. Scholz |
| *Anisantha tectorum* (L.) Nevski | *Hordeum vulgare* L. | *Rumex conglomeratus* Murray |
| *Arundo donax* L. | *Hormuzakia aggregata* (Lehm.) Guşul. | *Sagina apetala* Ard. |
| *Asteriscus aquaticus* (L.) Less. | *Hymenocarpos circinnatus* (L.) Savi | *Sagina maritima* Don |
| *Avena sterilis* L. | *Hyoscyamus albus* L. | *Salvia viridis* L. |
| *Cakile maritima* Scop. | *Lagurus ovatus* L. | *Sarcopoterium spinosum* (L.) Spach |
| *Campanula stellaris* Boiss. | *Lampranthus multiradiatus* (Jacq.) N.E.Br.* | *Senecio × berythaeus* A.Camus & Gomb. |
| *Capparis sicula* Veill. | *Lantana camara* L.* | *Sideritis romana* L. |
| *Cardopatium corymbosum* (L.) Pers. | *Leucaena leucocephala* (Lam.) de Wit* | *Silene aegyptiaca* (L.) L. |
| *Carissa macrocarpa* (Eckl.) A.DC.* | *Limbarda crithmoides* (L.) Dumort. | *Silene colorata* Poir. |
| *Carpobrotus edulis* (L.) N.E.Br.* | *Limonium mouterdei* Domina, Erben & Raimondo | *Silybum Marianum* (L.) Gaertn. |
| *Carthamus tenuis* (Boiss. & C. I. Blanche) Bornm. | *Limonium postii* Domina, Erben & Raimondo | *Sisymbrium officinale* (L.) Scop. |
| *Centaurea procurrens* Spreng. | *Limonium virgatum* (Willd.) Fourr. | *Sonchus oleraceus* L. |
| *Cerastium glomeratum* Thuill. | *Lotus angustissimus* L. | *Sphagneticola trilobata* (L.) Pruski* |
| *Cichorium pumilum* Jacq. | *Lotus cytisoides* L. | *Sporobolus pungens* (Schreb.) Kunth |
| *Convolvulus secundus* Desr. | *Lotus halophilus* Boiss. & Spruner | *Strelitzia reginae* Banks* |
| *Cota palaestina* Kotschy | *Lotus edulis* L. | *Thymbra capitata* (L.) Cav. |
| *Crepis* *aculeata* (DC.) Boiss. | *Lycopersicon esculentum* Mill.* | *Thymelaea hirsuta* (L.) Endl. |
| *Crepis palaestina* (Boiss.) Bornm. | *Malva oxyloba* Boiss. | *Tordylium trachycarpum* (Boiss.) Al-Eisawi |
| *Crithmum maritimum* L. | *Malva* sp. | *Tragopogon porrifolius L.* |
| *Crucianella aegyptiaca* L. | *Medicago littoralis* Loisel. | *Trifolium glanduliferum Boiss.* |
| *Cuscuta epithymum* (L.) L. | *Mercurialis annua* L. | *Trifolium purpureum* Loisel. |
| *Cyclamen persicum* Mill. | *Ochlopoa annua* (L.) H. Scholz | *Trifolium resupinatum* L. |
| *Cynodon dactylon* (L.) Pers. | *Onobrychis crista-galli* (L.) Lam. | *Trifolium scabrum* L. |
| *Cyperus rotundus* L. | *Orobanche nana* (Reut.) Beck | *Umbilicus intermedius* Boiss. |
| *Dactyloctenium aegyptium* (L.) Willd. | *Oxalis pes-caprae* L.* | *Urospermum picroides* (L.) F. W. Schmidt |
| *Daucus carota* L. | *Pancratium maritimum* L. | *Valantia muralis* L. |
| *Digitaria sanguinalis* (L.) Scop. | *Parapholis incurva* (L.) C. E. Hubb. | *Verbascum sinuatum* L. |
| *Dittrichia viscosa* (L.) Greuter | *Parietaria judaica* L. | *Veronica cymbalaria* Bodard |
| *Echium angustifolium* Mill. | *Paronychia argentea* Lam. | *Washingtonia* sp.* |
| *Elytrigia juncea* (L.) Nevski | *Phagnalon rupestre* (L.) DC. | *Yucca gigantea* Lem.* |
| *Epilobium tetragonum* L. |  |  |

Table 3. TWINSPAN analysis of floristic data set collected in Ras Beirut (Quadrat groups: F-A to F-Q, (number of quadrats), Alphabetical naming of quadrat groups by floristic and life fom classification are not related.).

|  |  | F-A  (4) | F-B  (1) | F-C  (2) | F-D  (6) | F-E (13) | F-F  (1) | F-G (8) | F-H (1) | F-I (22) | F-J (9) | F-K (1) | F-L (2) | F-M (1) | F-N (4) | F-O (1) | F-P (1) | F-Q (1) |
| --- | --- | --- | --- | --- | --- | --- | --- | --- | --- | --- | --- | --- | --- | --- | --- | --- | --- | --- |
| 1 | *Ficus microcarpa* L.f.* |  |  |  |  |  |  |  |  |  |  |  |  |  |  |  |  | VI 6 |
| 34 | *Piptatherum miliaceum* (L.) Coss. |  |  |  |  |  |  |  |  |  |  |  |  |  | III 4 |  |  | VI 2 |
| 100 | *Anagallis arvensis* L. |  |  | IV 2 | III 2 | III 1 |  |  |  | II 3 |  |  |  |  | IV 2 |  |  |  |
| 90 | *Veronica cymbalaria* Bodard |  |  | IV 5 |  |  |  |  |  |  |  |  |  |  | V 3 |  |  |  |
| 36 | *Cynodon dactylon* (L.) Pers. |  |  |  | II 1 | II 1 | VI 4 |  | VI 1 | II 1 |  |  |  | VI 3 | VI 5 | VI 3 | VI 2 |  |
| 35 | *Sporobolus pungens* (Schreb.) Kunth |  |  |  |  | II 1 |  |  |  |  |  |  |  |  | IV 6 |  |  |  |
| 6 | *Lantana camara* L.* |  |  |  |  | II 2 |  |  |  |  |  |  |  | VI 6 |  |  |  |  |
| 3 | *Carissa macrocarpa* (Eckl.) A.DC. |  |  |  |  |  |  |  |  |  |  |  |  |  |  | VI 6 |  |  |
| 79 | *Mercurialis annua* L. |  |  | IV 3 | V 3 | III 1 |  |  |  | II 1 |  |  |  | VI 3 | VI 3 |  |  |  |
| 18 | *Limonium mouterdei* Domina, Erben & Raimondo |  |  |  |  | V 3 |  |  |  | II 4 |  |  |  |  | IV 3 |  |  |  |
| 123 | *Malva oxyloba* Boiss. |  |  |  | II 1 | II 1 |  |  |  |  |  |  |  |  |  |  |  |  |
| 121 | *Sisymbrium officinale* (L.) Scop. |  | VI 2 |  | II 1 | III 2 |  | II 1 |  | II 1 |  |  |  |  |  |  |  |  |
| 114 | *Glebionis coronaria* (L.) Spach |  | VI 1 |  | III 2 | III 2 |  | II 1 |  | II 1 |  |  |  |  |  |  |  |  |
| 94 | *Aegilops geniculata* Roth |  |  | IV 2 |  |  |  |  |  |  |  |  |  |  |  |  |  |  |
| 91 | *Lagurus ovatus* L. |  |  |  | II 1 |  |  |  |  |  |  |  |  |  |  |  |  |  |
| 85 | *Onobrychis crista-galli* (L.) Lam. |  |  | IV 1 | II 1 |  |  |  |  |  |  |  |  |  |  |  |  |  |
| 83 | *Lotus edulis* L. |  |  |  |  | IV 2 |  |  |  |  |  |  |  |  |  |  |  |  |
| 82 | *Lotus halophilus* Boiss. & Spruner |  |  |  | II 1 | V 3 |  |  |  | II 1 | II 2 |  |  |  |  |  |  |  |
| 80 | *Hymenocarpos circinnatus* (L.) Savi |  |  | IV 3 |  |  |  |  |  |  |  |  |  |  |  |  |  |  |
| 77 | *Silene aegyptiaca* (L.) L. |  |  |  |  | II 1 |  |  |  |  |  |  |  |  |  |  |  |  |
| 76 | *Cakile maritima* Scop. |  |  |  | II 3 |  |  |  |  | II 1 |  |  |  |  |  |  |  |  |
| 72 | *Sagina apetala* Ard. |  |  |  | II 2 |  |  |  |  |  |  |  |  |  |  |  |  |  |
| 70 | *Cerastium glomeratum* Thuill. |  |  |  | III 2 |  |  |  |  |  |  |  |  |  |  |  |  |  |
| 62 | *Phleum subulatum* (Savi) Asch. & Graebn. |  |  |  | II 2 |  |  |  |  |  |  |  |  |  |  |  |  |  |
| 58 | *Pancratium maritimum* L. |  |  |  | II 2 | II 2 |  |  |  |  |  |  |  |  |  |  |  |  |
| 55 | Anacamptis sancta (L.) R. M. Bateman |  |  |  | II 3 |  |  |  |  |  |  |  |  |  |  |  |  |  |
| 52 | *Daucus carota* L. |  |  | IV 1 |  |  |  |  |  |  |  |  |  |  |  |  |  |  |
| 49 | *Anchusa hybrida* Ten. |  |  |  | IV 2 | IV 3 |  |  |  |  |  |  |  |  |  |  |  |  |
| 47 | *Silybum Marianum* (L.) Gaertn. |  |  | IV 1 |  |  |  |  |  |  |  |  |  |  |  |  |  |  |
| 44 | *Alcea setosa* (Boiss.) Alef. |  |  | IV 4 | V 3 | II 2 |  |  |  |  |  |  |  |  |  |  |  |  |
| 42 | *Polygonum equisetiforme* Sm. |  |  | IV 6 |  |  |  |  |  |  |  |  |  |  |  |  |  |  |
| 40 | *Paronychia argentea* Lam. |  |  | IV 2 | II 2 |  |  |  |  |  |  |  |  |  |  |  |  |  |
| 28 | *Convolvulus secundus* Desr. |  |  |  | III 6 |  |  |  |  |  |  |  |  |  |  |  |  |  |
| 25 | *Phagnalon rupestre* (L.) DC. |  |  |  | II 3 |  |  |  |  |  |  |  |  |  |  |  |  |  |
| 23 | *Limonium postii* Domina, Erben & Raimondo |  |  |  |  | III 4 |  |  |  |  |  |  |  |  |  |  |  |  |
| 20 | *Dittrichia viscosa* (L.) Greuter |  |  |  | IV 5 | II 4 |  |  |  | II 1 |  |  |  |  |  |  |  |  |
| 5 | *Thymelaea hirsuta* (L.) Endl. | III 5 |  |  | II 6 | II 5 |  |  |  |  |  |  |  |  |  |  |  |  |
| 117 | *Tordylium trachycarpum* (Boiss.) Al-Eisawi |  |  | IV 1 | IV 3 |  |  |  |  | II 2 |  |  | IV 1 |  |  |  |  |  |
| 111 | *Senecio × berythaeus* A.Camus & Gomb. |  |  | IV 2 | II 1 | III 1 |  |  |  | II 2 | III 1 |  |  |  |  |  |  |  |
| 107 | *Cota palaestina* Kotschy |  | VI 1 |  |  | V 1 |  |  |  | II 2 | III 1 |  |  |  |  |  |  |  |
| 97 | *Avena sterilis* L. |  | VI 1 | IV 6 |  |  |  |  |  | II 2 | II 1 |  |  |  |  |  |  |  |
| 96 | *Anisantha rigida* (Roth) Hyl. |  |  | IV 2 | II 2 |  |  |  |  | II 2 | II 1 |  |  |  |  |  |  |  |
| 78 | *Silene colorata* Poir. |  |  |  |  | II 2 |  |  |  |  | II 1 |  |  |  |  |  |  |  |
| 48 | *Tragopogon porrifolius* L. |  |  | IV 1 | II 2 |  |  | II 1 |  |  |  |  |  |  |  |  |  |  |
| 45 | *Verbascum sinuatum* L. |  |  |  | III 4 | II 1 |  |  |  | II 2 |  |  |  |  |  |  |  |  |
| 21 | *Helichrysum stoechas* (L.) Moench | III 6 |  | IV 4 | III 3 |  |  |  |  | II 5 |  |  |  |  |  |  |  |  |
| 118 | *Carthamus tenuis* (Boiss. & C. I. Blanche) Bornm. | III 1 | VI 3 | IV 2 |  | II 1 |  |  |  | II 1 |  |  |  |  |  |  |  |  |
| 110 | *Picris rhagadioloides* (L.) Desf. | III 1 | VI 2 |  |  |  |  |  |  |  |  |  |  |  |  |  |  |  |
| 109 | *Hedypnois rhagadioloides* (L.) F. W. Schmidt |  | VI 1 |  |  |  |  |  |  |  |  |  |  |  |  |  |  |  |
| 92 | *Crucianella aegyptiaca* L. |  | VI 1 |  |  |  |  |  |  |  |  |  |  |  |  |  |  |  |
| 88 | *Salvia viridis* L. |  | VI 3 |  |  |  |  |  |  |  |  |  |  |  |  |  |  |  |
| 87 | *Trifolium purpureum* Loisel. |  | VI 1 |  |  |  |  |  |  |  |  |  |  |  |  |  |  |  |
| 54 | *Cyclamen persicum* Mill. |  | VI 1 |  |  |  |  |  |  |  |  |  |  |  |  |  |  |  |
| 46 | *Cardopatium corymbosum* (L.) Pers. |  | VI 2 |  |  |  |  |  |  |  |  |  |  |  |  |  |  |  |
| 16 | *Sarcopoterium spinosum* (L.) Spach |  | VI 5 |  |  |  |  |  |  |  |  |  |  |  |  |  |  |  |
| 15 | *Thymbra capitata* (L.) Cav. | VI 5 | VI 2 |  |  | II 3 |  |  | VI 3 |  |  |  |  |  |  |  |  |  |
| 56 | *Oxalis pes-caprae* L.* |  |  |  |  |  |  |  |  | II 2 |  |  |  |  | III 6 |  |  |  |
| 4 | *Pittosporum tobira* (Thunb.) W. T. Aiton* |  |  |  |  |  |  |  |  | II 5 |  |  |  |  |  |  | VI 6 |  |
| 112 | *Crepis palaestina* (Boiss.) Bornm. |  | VI 1 | IV 3 | II 1 | III 2 |  | II 1 |  | II 2 | III 3 |  |  |  |  |  |  |  |
| 75 | *Valantia muralis* L. |  |  |  | II 1 | III 1 |  |  |  | III 1 | II 1 |  | IV 2 |  |  |  |  |  |
| 71 | *Polycarpon tetraphyllum* (L.) L. |  |  | IV 3 | V 3 | II 1 | VI 2 | III 1 |  | III 2 | IV 2 | VI 1 |  |  |  |  | VI 1 |  |
| 29 | *Lotus cytisoides* L. | III 2 |  |  |  |  |  |  | VI 2 |  | II 4 |  | IV 5 |  |  |  |  |  |
| 122 | *Malva* sp. |  |  |  |  | II 1 |  |  |  | II 2 |  |  |  |  |  |  |  |  |
| 108 | *Crepis* *aculeata* (DC.) Boiss. |  |  |  | V 2 | II 2 |  |  |  | III 3 | IV 2 |  |  |  |  |  |  |  |
| 105 | *Asteriscus aquaticus* (L.) Less. |  | VI 1 |  |  |  |  |  | VI 3 |  |  |  | IV 2 |  |  |  |  |  |
| 74 | *Galium murale* (L.) All. |  |  |  | III 4 |  |  |  |  |  | IV 2 | VI 2 | IV 2 |  |  |  |  |  |
| 57 | *Umbilicus intermedius* Boiss. | III 2 |  |  |  |  |  |  |  | II 2 |  |  |  |  |  |  |  |  |
| 39 | *Elytrigia juncea* (L.) Nevski | III 2 | VI 1 | IV 1 | II 1 |  |  |  | VI 2 | II 2 |  |  |  |  |  |  |  |  |
| 30 | *Capparis sicula* Veill. |  |  |  |  | II 1 |  |  |  | II 2 |  |  |  |  |  |  |  |  |
| 24 | *Limonium virgatum* (Willd.) Fourr. | III 3 |  |  |  |  |  |  | VI 5 |  |  |  |  |  |  |  |  |  |
| 104 | *Plantago lagopus* L. |  |  |  | II 1 |  |  |  |  |  | III 3 |  |  |  |  |  |  |  |
| 103 | *Plantago coronopus* L. |  |  |  |  |  |  |  |  | II 1 | IV 3 |  | IV 5 |  |  |  |  |  |
| 95 | *Anisantha tectorum* (L.) Nevski |  |  |  |  |  |  |  |  | II 1 | III 1 |  |  |  |  |  |  |  |
| 86 | *Trifolium glanduliferum* Boiss. |  |  |  |  |  |  |  |  |  | II 1 |  |  |  |  |  |  |  |
| 73 | *Sagina maritima* Don |  |  |  |  |  |  |  |  | II 1 | IV 2 |  |  |  |  |  |  |  |
| 66 | *Dactyloctenium aegyptium* (L.) Willd. |  |  |  |  |  |  |  |  | II 1 | III 3 |  |  |  |  |  |  |  |
| 65 | *Trifolium resupinatum* L. |  |  |  |  |  |  |  |  |  |  |  | IV 1 |  |  |  |  |  |
| 64 | *Trifolium scabrum* L. |  |  |  |  |  |  |  |  |  | III 1 |  |  |  |  |  |  |  |
| 63 | *Rostraria smyrnacea* (Trin.) H. Scholz | III 2 |  |  | III 2 |  |  |  |  | II 1 | IV 2 |  | IV 1 |  |  |  |  |  |
| 61 | *Parapholis incurva* (L.) C. E. Hubb. |  |  |  |  |  |  |  |  |  | III 2 |  |  |  |  |  |  |  |
| 50 | *Rumex conglomeratus* Murray |  |  |  |  |  |  |  |  |  | II 1 |  |  |  |  |  |  |  |
| 41 | *Phyla nodiflora* (L.) Greene* |  |  |  |  |  |  |  |  | I 2 | IV 5 |  |  |  |  |  |  |  |
| 26 | *Echium angustifolium* Mill. |  |  |  |  |  |  |  |  |  | III 6 |  |  |  |  |  |  |  |
| 19 | *Crithmum maritimum* L. |  |  |  |  |  |  | II 2 |  | II 4 |  |  | VI 6 |  |  |  |  |  |
| 13 | *Ricinus communis* L.* |  |  |  |  |  |  |  |  |  |  | VI 3 |  |  |  |  |  |  |
| 7 | *Ficus carica* L. |  |  |  |  |  |  |  |  |  |  | VI 6 |  |  |  |  |  |  |
| 120 | *Urospermum picroides* (L.) F. W. Schmidt |  |  |  | II 3 | II 1 |  | IV 2 |  | II 1 | V 2 |  |  |  |  |  |  |  |
| 59 | *Cyperus rotundus* L. |  |  |  |  |  | VI 3 | III 3 |  |  | III 1 |  |  |  |  |  |  |  |
| 81 | *Lotus angustissimus* L. |  |  |  |  |  |  |  |  | II 3 | III 1 |  |  |  |  |  |  |  |
| 51 | *Parietaria judaica* L. | III 2 |  |  |  |  |  |  |  | III 3 | II 3 | VI 3 |  |  |  |  |  |  |
| 31 | *Matthiola crassifolia* Boiss. & Gaill. | IV 4 | VI 3 |  | VI 4 | V 2 |  | VI 4 | VI 2 | VI 4 | V 4 |  | IV 3 |  |  |  |  |  |
| 119 | *Sonchus oleraceus* L. |  |  |  |  |  |  | III 1 |  | II 1 | II 1 |  |  |  |  |  |  |  |
| 116 | *Hormuzakia aggregata* (Lehm.) Guşul. |  |  |  |  |  |  |  |  | II 3 |  |  |  |  |  |  |  |  |
| 113 | *Erigeron bonariensis* L.* |  |  |  |  |  |  | VI 1 |  |  |  |  |  |  |  |  |  |  |
| 99 | *Hyoscyamus albus* L. |  |  |  |  |  |  |  |  | II 3 |  |  |  |  |  |  |  |  |
| 98 | *Hordeum vulgare* L. |  |  |  |  |  |  |  |  | II 2 |  |  |  |  |  |  |  |  |
| 84 | *Medicago littoralis* Loisel. |  |  |  |  |  |  |  |  | II 2 |  |  |  |  |  |  |  |  |
| 69 | *Campanula stellaris* Boiss. |  |  |  |  |  |  |  |  | II 2 |  |  |  |  |  |  |  |  |
| 68 | *Alyssum strigosum* Banks & Sol. |  |  |  |  |  |  |  |  | II 2 |  |  |  |  |  |  |  |  |
| 53 | *Erigeron canadensis* L.* |  |  |  |  |  |  | III 1 |  | II 1 |  |  |  |  |  |  |  |  |
| 38 | *Carpobrotus edulis* (L.) N.E.Br.* |  |  |  |  |  |  |  |  | II 6 |  |  |  |  |  |  |  |  |
| 37 | *Lampranthus multiradiatus* (Jacq.) N.E.Br.* |  |  |  |  |  |  | V 6 |  |  |  |  |  |  |  |  |  |  |
| 33 | *Centaurea procurrens* Spreng. |  |  |  |  |  |  |  |  | II 1 |  |  |  |  |  |  |  |  |
| 27 | *Sphagneticola trilobata* (L.) Pruski* |  |  |  |  |  | VI 6 |  |  | II 2 |  |  |  |  |  |  |  |  |
| 22 | *Limbarda crithmoides* (L.) Dumort. |  |  |  |  |  |  | II 2 | VI 5 | II 2 |  |  |  |  |  |  |  |  |
| 17 | *Galium canum* DC. |  |  |  |  |  |  |  |  | III 4 |  |  |  |  |  |  |  |  |
| 14 | *Arundo donax* L. |  |  |  |  |  |  |  |  | II 6 |  |  |  |  |  |  |  |  |
| 12 | *Strelitzia reginae* Banks* |  |  |  |  |  |  | III 4 |  |  |  |  |  |  |  |  |  |  |
| 11 | *Washingtonia* sp.* |  |  |  |  |  |  | III 4 |  |  |  |  |  |  |  |  |  |  |
| 10 | *Yucca gigantea* Lem.* |  |  |  |  |  |  | II 5 |  |  |  |  |  |  |  |  |  |  |
| 9 | *Agave attenuata* Salm-Dyck* |  |  |  |  |  |  | III 4 |  |  |  |  |  |  |  |  |  |  |
| 8 | *Agave americana* L.* |  |  |  |  |  |  | III 6 |  |  |  |  |  |  |  |  |  |  |

The Roman number corresponds to species constancy within each TWINSPAN group (I = 5% or less; II = 6 – 20%; III = 21 – 40%; IV = 41 – 60%; V = 61 – 80%; VI = 81 – 100%). The Arabic number indicates average species abundance for each group on the Domin scale (1 = less than 1%; 2 = 1 – 4%; 3 = 5 – 10%; 4 = 11 – 25%; 5 = 26 – 50%; 6 = 51 – 100%). .
