# Supplementary material for: Biodiversity conservation in cities: Defining habitat analogues for plant species of conservation interest
Source: PLoS One. 2020 Jun 9;15(6):e0220355. doi: 10.1371/journal.pone.0220355 (PMC7282666; doi:10.1371/journal.pone.0220355)
Supplement: Attachment — Submitted filename: response to reviewers form feb 29 2020 SNT.docx [file pone.0220355.s007.docx]

 5. Review Comments to the Author

Please use the space provided to explain your answers to the questions above. You may also include additional comments for the author, including concerns about dual publication, research ethics, or publication ethics. (Please upload your review as an attachment if it exceeds 20,000 characters)

PONE-D-19-19705
Biodiversity conservation in cities: Defining habitat analogs for plant species of conservation interest
PLOS ONE

*Thank you for this opportunity we present a revised version of the manuscript which addresses all points raised during the review process. As instructed below please find responses listed under each comment.*

Reviewer #1:

1. The manuscript includes a novel stepwise approach in identifying habitats suitable for target species. The use of a combination of lifeform spectrum and physiognomic characteristic and floristic identity has helped in proposing management strategies for Matthiola crassifolia. I admire the approach

*We are grateful for the positive feedback of Reviewer #1 regarding the approach we have adopted*

Reviewer #2:

INTRODUCTION

1. The introduction is missing citations, as many statements are not supported by literature.

*Major revisions were made to the introduction to better support statements by literature. At least twenty eight new references were added including the ones proposed by the reviewer.*

2. I would only use the world “artificial” (e.g. line 76) to describe urban habitats that have been deliberately designed by man with a specific purpose. Not all urban habitats should be classified as such.

*The table below lists all the places where the word artificial was removed as suggested by the reviewer.*

| Line/Table | Modification |
| --- | --- |
| 53 | Not removed because urban habitat |
| 74 | Removed |
| 76 | Removed |
| 78 | Removed |
| Table 8 | Not removed because urban habitat |
| Table 8 | Not removed because urban habitat |

3. Before introducing Table 1, the authors mention that current data collection methods are designed for non-urban contexts. However, they should be aware that very important work has been done to classify the urban habitats inside the city of Berlin. This project lasted several years and was done by the Senate Department for Urban Development and Housing of Berlin (more info here: <http://www.stadtentwicklung.berlin.de/umwelt/umweltatlas/ed508_03.htm>). They differentiated more than 7000 different urban biotopes/habitats inside the city of Berlin.

*A section was added in the introduction to highlight this study and explain why the collection method is not applicable to Beirut*

4. I would include in the introduction an hypothesis stating what kind of analogue habitats the authors expect to find for M. crassifolia.

*As recommended by the reviewer a hypothesis was added as a section before the objective*

5. In general, I have the feeling that this section could profit from a deeper insight into current findings in urban ecology. This is a non-exhaustive list of some important literature that could help:

*All of the literature suggested by the reviewer, except Chocholouskova et al. 2003 has been added to the introduction.*

METHODS

6. From my perspective, the description of the genus and the whole range of species beyond M. crassifolia is not necessary.

*The section describing the genus and the whole range of species was deleted as suggested (lines 114-119) also reference 43 was deleted.*

7. In lines 131 to 135, I would state that the mentioned sites are cities.

*Lines 131 to 135 were modified to indicate that the sites are in cities*

Many readers might not be familiar with the geography of Lebanon. Additionally, I would use the English instead of the local name of the city (i.e. Sidon instead of Saida) for international readers.

*A brief description of the geography of Lebanon was added as suggested by the reviewer in the methodology section under study location. All names of locations were changed to English as suggested by the reviewer.*

8. I don’t fully understand what the authors wanted to achieve with point 3 starting in line 170. Where was the second quadrat placed and why?

*In point 3 we wanted to explain further our sampling strategy, specifically how we selected the quadrat location because we faced challenges in the field when placing quadrats and felt that this information is worth sharing. We revised the text to read as follows:*

*“We made three modifications to the sampling technique to address site-specific challenges; 1) When the boundary of a plant assemblage was not clearly defined due to site disturbance, we placed quadrats in locations where the target species was highly represented and at least one quadrat where the target species was minimally represented assuming that this location constitutes the boundary of the sampled plant assemblage. 2) When species had an ‘individualistic’ distribution pattern, we increased the number of quadrats to capture the observed variation and compensate for the difficulty to define the boundaries of the plant assemblage [60], 3) When the target species was consistently not present in a given vegetation assemblage, we placed one quadrat. “*

RESULTS

9. I would argue that this section in general needs to be easier to interpret and more visual. I think most tables don’t necessarily need to be included in the results. Perhaps I would include them as an annex and only represent a summary of some of them in the results. For example, Table 2 could be summarized showing only the genus that co-occur with M. crassifolia, not all the species, or Table 4 could be easily summarized with a pie chart showing the different proportion of each life-form. Additionally, some data mentioned in the text could be represented visually in a graph or similar (i.e. the proportion of M. crassifolia found in remnant sites vs. anthropogenic areas, or a map showing the location of these 20 sites where M. crassifolia was lost)

*As per reviewer’s suggestion we simplified this section by deleting table 2 and table 3 from the manuscript and included them in the supplementary material section. We also simplified Table 4 and we added a map to visually show the locations of the 20 sites.*

DISCUSSION

10. I think the authors should discuss about their own results earlier in this section. Perhaps some of the text between lines 353-396 could go to the introduction.

*As suggested by the reviewer we moved the section between lines 353 and 396 to the introduction and started discussing our own results early in the section.*

11. It could be interesting to include some discussion about the characteristics of the sites where M. crassifolia was lost.

12. I think it could be interesting to discuss about the ecological reasons explaining why there is a difference in the presence of M. crassifolia in remnant sites vs. anthropogenic sites.

*As suggested by the reviewer a description of the sites were M. crassifolia was lost was added as follows:*

*“At the end of the four-year study, M. crassifolia was no longer seen in 16 out of 73 sites. Causes for this loss may be due to: the landscape maintenance practices (gardeners consider M. crassifolia as a weed and remove it purposely), or the landscape plant compositions which create conditions that exclude M. crassifolia; street medians are planted with evergreen peat accumulating groundcovers and shade producing shrubs. In contrast, in sites where M. crassifolia is still present gardeners remove plants during their dieback stage, which includes seed-bearing silique fruits, but they keep seedlings and flowering plants. Furthermore, in these sites, street medians have unplanted areas and bare ground with partial cover by reptant succulents.”*

13. Regarding the non-random design of this study, it might be interesting if the authors would consider this paper when interpreting their results: Diekmann, M., Kühne, A. & Isermann, M. Folia Geobot (2007) 42: 179. <https://doi.org/10.1007/BF02893884>. It shows that in small plot sizes like the ones used in this study, non-randomly placed plots result in general in higher species richness than if they were randomly distributed.

*Findings by Diekmann et al were considered in the methodology section and the discussion section.*

14. As a general comment for this section, I would argue that in order to develop a methodology to assess the habitat analogues of a species, the authors should consider including an assessment of not only floristics and physiognomy, but also of the local habitat conditions. As an example, we could reflect on the areas where M. crassifolia thrives (those with palms, succulents or dry shrubs) - perhaps there is something in these locations beyond the species assemblages that can explain why the target species is performing favorably. Are these locations the least managed? Are they in spontaneous urban wastelands? I think it is very important that the authors include an additional assessment of the local habitat conditions in their study.

*As suggested by the reviewer description of local habitat conditions were incorporated in the table. In addition, the following was added to the text.*

*“The habitat conditions in quadrat groups of high representation of the target species were not the same and reflected a wide range of potential habitat analogues for M. crassifolia. These varied from abandoned buildings to highly managed street medians.”*

GENERAL REMARKS

I think more work is needed to improve the scientific quality of this paper. The authors should make use of the broad literature available in the field of urban ecology and their methodology should include an assessment of the local habitat conditions when assessing habitat analogues for a species of conservation concern.

*As indicated above a large number of references were consulted and incorporated to improve the scientific quality of the paper.*

Reviewer #3: Dear authors of „Biodiversity conservation in cities: Defining habitat analogs for plant species of conservation interest“,

The idea of identifying habitat analogs for species of conservation interest within human-dominated landscapes is an interesting idea for improving species conservation and, in your case, for improving the contribution of urban areas to the protection of biological diversity.
After reviewing your manuscript, I have a number of concerns that in my opinion should be solved before the manuscript is ready for publication. I will describe my concerns below, point by point.

*We thank the reviewer for his positive feedback.*

Abstract:

- Line 28: I am not sure what you want to express with the first sentence of the abstract. Do you want to express that urban environmental conditions change the composition of species assemblages or that species in urban areas interact differently with each other than they do within non-urban habitats? If you mean the latter, is there proof for it? For example, would two species interact differently with each other depending on whether they occur in an urban or in a non-urban area?

*In the first sentence of the abstract we mean that urban environmental conditions change the composition of species assemblages. The sentence has been changed to reflect this meaning*

*“Urban conditions have become primary drivers of species interactions that impact the composition of species assemblages.”*

- You distinguished among native, naturalized, ornamental garden escapes and invasive plant species (e.g., lines 29/30, 73, 145). Could you state more precisely that these are native and non-native species, with the three latter groups comprising non-native species?

*As suggested by the reviewer sentences were modified to clarify that these are native and non-native species, with the three latter groups comprising non-native species*

*Lines 29/30: They consist of managed vegetation and spontaneous assemblages of native, naturalized, casual non-native, and invasive species.*

*Line 73 was removed.*

*Line 145: Paul Mouterde, who conducted floristic studies in Beirut in the 20th century, reported 1200 floral species including native and non-native species [53].*

Also, I’d rather talk about casual non-native species than about ornamental garden escapes because usually, non-naturalized non-native species in urban areas do not only (although to a large share) include ornamental garden escapes but also other introduced species that have not naturalized (yet), such as species accidentally introduced.

*As per reviewer suggestion we omitted the term ornamental garden escapes and replaced it with casual non-native species*

- Line 34: Where are these 12 study sites located? Please say so in the abstract and consider adding a figure to the manuscript that depicts the location of sites.

*We added a sentence to the abstract to explain where the study locations are and a figure map was added to show the location of the sites*

Introduction: The introduction would benefit from better linking it to recent urban biodiversity research, especially to our knowledge about species conservation in urban areas, to the concept of novel ecosystems, and to functional traits and functional groups:

- The persistence of species of conservation interest within urban areas is not in general unlikely (and thus I do not completely agree with your sentence in lines 75/76). A range of threatened species can occur within urban areas (see Ives et al. 2016, Global Ecology and Biogeography 25, 117-126). Whether a species will be able to persist within urban environmental conditions does depend on its functional traits (cf. Williams et al. 2015, Perspectives in Plant Ecology, Evolution and Systematics 17, 78–86).

*As suggested by the reviewer, the following was added to the introduction:*

*“Urban habitats tend to favor the persistence of plant species with particular trait combinations that appear well suited to the conditions (Duncan et al, 2011). Furthermore, certain plant functional traits tend to increase in response to urbanisation, while other traits have mixed responses (Williams et al 2015). For example, it has been shown that urbanized grid cells favor wind pollinated plants, plants with scleromorphic leaves or plants dispersed by animals on insect pollinated plants, plants with hygromorphic leaves or plants dispersed by wind than other grid cells (Knapp, 2010). Also, Acidophiles might already disappeared from urban areas (Knapp et al. 2009). Thus, identifying predictable relationships between plant traits and environmental conditions or disturbances is a promising approach for understanding how plant communities change in response to human land-use modification (Westoby & Wright, 2006; Díaz et al., 2007).* 

- Introduction, lines 73/74: Well-established standard classifications of urban habitats exist, such as the four types of urban nature by Kowarik (1992 – cited e.g. in Kowarik 2018 Urban Forestry & Urban Greening 29, 336-347). You do not necessarily need to refer to one of these classifications, but please note that urban areas usually not only comprise artificial habitats but also remnants of natural habitats.

*As suggested by the reviewer the literature was consulted and the information incorporated as per below:*

*“Novel ecosystems are human-modified ecosystems that have likely been irreversibly altered by intense impacts on abiotic conditions or biotic composition (Planchuelo, G., von Der Lippe, M., & Kowarik, I. (2019); Hobbs, R. J.,et al. (2009); Kowarik & von der Lippe, 2018). Among these novel ecosystems are Urban green spaces that include both nonnative vegetation assemblages and remnants of natural habitats consisting of native, spontaneous, naturalized, and invasive species (Rupprecht, C. D., Byrne, J. A., Garden, J. G., & Hero, J. M., 2015). These spaces are often unintentionally managed and contain a range of both early- and late-succession vegetation.”*

- Lines 84-86: I do not see why the methods used to describe vegetation that you present should only work in natural areas. I guess that what you want to express is that the TWINSPAN-classification of groups does not work that well because, as you wrote later, there are many unique co-occurrences of plant species at different urban sites. However, the methods you present in Table 1 can be applied in all kinds of terrestrial plant communities, including urban ones. It’s just that the results differ among urban and non-urban habitats.

*This text was removed because Table 1 was deleted as suggested by reviewer #2.*

Concerning the many unique co-occurrences of plant species at different urban sites, I suggest that you refer to the concept of novel ecosystems (see Hobbs et al. 2006, Global Ecology and Biogeography 15, 1-7 for the overall concept and Kowarik 2011, Environmental Pollution 159, 1974-1983 for a review on novel urban ecosystems) because what you describe – untypical, unique species assemblages – might exactly be this – novel urban ecosystems.

*As suggested by the reviwer, we used novel ecosystems in the introduction* to describe the occurrence of *untypical, unique species assemblages.*

- Table 1: How did you collect / choose these methods? Did you perform some kind of literature review to do so? If not, how do you know that the overview is complete? Also, I do not see much use in presenting all this detailed information. The table occupies a lot of space but the basic message could be wrapped into one or two sentences: The methods you present in Table 1 describe vegetation based on species identity and abundance, species functional traits, structural characteristics or degree of naturalness. All other details are not necessary for understanding your study and interested readers can refer to the literature cited. So, my suggestion is to delete Table 1 and to wrap its most important content into one or two sentences with references. (cf. Discussion, line 353 where you wrote about “two main vegetation description methods” – so, this can be nicely wrapped)

*Table 1 was deleted and references incorporated in a section as shown below and as suggested by the reviewer*

*“There are various methods that describe vegetation based on species identity and abundance, species functional traits, structural characteristics or degree of naturalness. Floristic surveys are one of two main vegetation description methods used to obtain baseline data on native species of conservation interest and to generate community classification schemes and structure patterns that vary predictably in response to external factors such as environmental stress and disturbance [18]. Floristic method uses taxonomic identification and species abundance to describe vegetation. From the perspective of floristics, plant species found in an area are unique and capable of coexisting as distinct, recognizable units that are repeated regularly in response to biotic and environmental variations [67, 68, 69, 70, 71]. The other method, physiognomy, describes vegetation according to external morphology, life form, stratification, and size of each species [8, 9, 10, 11, 12, 13, 14, 15, 16, 17]. More recently, a method, EcoVeg, has been developed that combines physiognomic, floristic and ecological descriptors of a vegetation and applies different rationales depending on whether the vegetation natural or cultural [19].”*

- Citations in lines 94/95 – a lot. Maybe it is not necessary to cite all of these 17 references. Rather choose some and provide them as example references.

*As suggested by the reviewer we removed six references and briefly described the remaining ones in the text.*

- Lines 95/96: please, do provide references for the statement that physiognomic and structural vegetation description may be a more useful tool for urban biodiversity than floristics. To better link your manuscript to recent urban biodiversity research, I think it is useful to refer to functional plant traits. The physiognomic characteristics that you look at equal functional traits and functional groups. (See e.g. Williams et al. 2015, Perspectives in Plant Ecology, Evolution and Systematics 17, 78–86 and Lavorel et al. 1997, Trend in Ecology and Evolution 12, 474-478). Especially, to relate species to environmental conditions or their ability to co-occur with other species, trait-based approaches (or physiognomic approaches, as you call them) are indeed more helpful than floristic approaches.

*As suggested by the reviewer, references were incorporated to support the statement that physiognomy and structural vegetation description may be more useful tool for urban biodiversity than floristics. We also referred to functional plant traits as suggested. These were added in the introduction and in the discussion section.*

In addition, some information on the target species and why it is the target species of your study should be added to the Introduction. From all the data that you present, I got the impression that Matthiola crassifolia performs reasonably well in the urban area of Beirut. So, is it really threatened by urbanization? Also, in which natural habitats does the target species usually occur? Please, add this information to the description of the study species as it seems basic to understanding habitat analogs (as these will be analog to the natural habitats of the target species, won’t they?)

*As suggested by the reviewer we added in the introduction information on the target species, why it is the target species, and information about its conservation status and natural habitats. We explained that the population size and number are decreasing.*

Materials and Methods:
The identification of habitat analogues for the target species is solely based on the identification of species and life form types it occurs with. No abiotic conditions (temperature, soil type, soil moisture, pH, nutrients, surrounding land cover, …) have been taken into account although the occurrence and abundance of a species is not only determined by its biotic interactions but also, to a major extent, by abiotic conditions. Why haven’t abiotic conditions been taken into account?

*We added the section below to explain why we decided not to measure abiotic factors.*

*“As the first meta‐analysis on intra‐urban biodiversity variation worldwide showed that patch area and corridors have the strongest positive effects on biodiversity, complemented by vegetation structure and that local, biotic and management habitat variables were found to be significantly more important than landscape, abiotic or design variables (Beninde, J., Veith, M., & Hochkirch, A., 2015), our study sought to identify habitat analogues for the target species primarily on the identification of species and life form types it occurs with.”*

- How were the 12 study sites selected? Was selection based on specific criteria? Which data (such as habitat or land-use maps) were used to select sites? I do understand the method that you used, still, it does not explain to me how sites were selected, i.e.: Were all anthropogenic and semi-natural habitats that occur in Ras Beirut taken into account or were specific anthropogenic and semi-natural habitats selected?

*As per reviewer suggestion, we added the text below to clarify the specific criteria that we followed to select the 12 study sites.*

*“Baseline data collection was performed three years before the start of the study to ensure comprehensive coverage of all informal green space locations. During 2012, all informal green spaces in the study location were surveyed to determine in which of these spaces M. crassifolia was present. In subsequent years (2013 and 2014) annual visits were made to all identified green spaces during flowering season of the target species, regardless of whether the species was present or not. In 2015, sites were selected according to management intensity of anthropogenic sites (high or low management intensity) and diversity of semi-natural habitats.*

This question is crucial because in the end, there are 78 plots and in 73 of these plots, the target species occurs. How can you, statistically sound, assess the preference of the target species for specific species assemblages if you have hardly any sites where it does not occur?

*Even though we had a limited number of quadrats in vegetation assemblages were the target species was consistently absent, we were able to discern the preference of the target species by analyzing a range of observed species abundance and its relation to vegetation assemblages where it occurs.*

- Basic temporal information is missing in the Materials and Methods chapter: How long did the study last? When did the study start? When did the study end?

*As indicated above baseline field information was collected between 2012 and 2014 and the study was conducted in 2015*

- Lines 122-124: Why are the species group names written in large letters?

*Typographic error was corrected to normal letter size as indicated by the reviewer*

- Line 127: add “should” among “species proposing that it” and “be considered”

*Modifications made as suggested by the reviewer*

- Line 133: “three out of five previously reported sites, Beirut and Byblos”. These are just two sites, not three. What’s the third one?

*The third location is Ras Beirut and was lumped under Beirut. The text was modified as follows to clarify the number of previously reported sites.*

*“The most comprehensive record of the distribution and status of M. crassifolia prior to this study was by [45] who performed a systematic survey of the Lebanese coast and recorded the presence of the species in three out of five previously reported sites, Ras Beirut, Beirut and Byblos. In this study, Ras Beirut and Beirut were considered a single locality.”*

- Lines 134/135: What about Amchit? Has it been extirpated there as well or does it still occur there?

*The following was added to the section where the species is described to address this inquiry*

*“Our field survey to these localities confirmed the extinction of M. crassifolia in Sidon and its continued presence in Khaldeh, Beirut, Amchit and Byblos [47].”*

- Lines 142: With 20 km², you refer to the city, not to the total metropolis of Beirut, right? Please specify.

*The sentence was modified to explain that the area refers to the city and not to greater Beirut*

- Lines 156: Is there a reference for the early botanical studies of semi natural areas in Beirut? Also, when did these studies take place?

*Yes there is a reference for the early botanical studies of semi natural areas in Beirut. This information was added in the study area section of the text.*

- Lines 157/158: Beirut as a type III-city according to Hahs et al. 2009: Hahs et al. refer to the year 1600 as a threshold (extensive transformations before or after 1600). As Beirut is a very old city, I guess that it might have experienced extensive transformations well before 1600. So, I am not convinced by your argument that Beirut is a type III-city – why not a type I-city? Please, be more precise in making your argument.

*More information was provided to explain why we consider Beirut as a type III city. The information includes the historical evolution of Beirut based on existing maps. Text and maps were added to the manuscript in the materials and methods section:*

*“The study location, Ras Beirut and the southern part of the promontory of Beirut, can be considered a type III city that is likely to be carrying an extinction debt because extensive landscape transformations occurred after initial floristic surveys (57). Although the expansion of the city started in 1840, the city passed through five stages of transformation whereby Ras Beirut and the southern part of the promontory was still semi-natural till 1943 (Yassin, 2010). The earliest botanical studies in the region took place in the mid-1800 (Boissier 1888) and continued giving considerable focus to Beirut and its environments till the 1930s (e.g. Mouterde 1935; Post & Dinsmore 1933)”.*

- Lines 180/181 University = American University of Beirut? Please, specify.

*American University of Beirut was spelled out as requested by the reviewer*

- Please, explain in more detail what TWINSPAN does, e.g. shortly mention what the cut levels are applied for. Also, please in the “Analysis” chapter, do explain how all this analysis is related to the target species. It is not mentioned at all how you identified habitat analogs for it.

*Definition of TWINSPAN was added, in the analysis section we explained what it does and how the analysis relates to the target species.*

*“The Two Way INdicator SPecies ANalysis (Twinspan*) (Hill, 1979a) is a method for classifying communities according to hierarchical divisions based on progressive refinement of a single ordination axis of a (sites × species) data matrix (Legendre & Legendre, 2012).”*

Results:
- Line 207: What does “800 m” stand for?

*This value was removed and replaced (see response to reviewer above) to 17out 73 sites.*

- I think that in addition to (or instead of) Tables 3 and 5, ordination plots will be illustrative to

*We explain in the materials and methods section that “ordination based on non-randomly based plots may be biased and we base our statement on a reference which we include in the text.*

The tables occupy a lot of space and are tedious to read.

*We modified the tables 3 and 5 and simplified them as suggested by the reviewer*

- Line 244: add “as” between “around evergreen ornamentals such” and “the”.

*Changes made as suggested by reviewer*

- Line 246: Change “florsitics” to “floristics”

*Changes made as suggested by reviewer*

- Table 4: Three different codes (Life-form eight digit name, Abbreviated lifeform category, and Numeric code) are used here for the different life forms but no description/ definition is provided. Please, choose one out of three codes and add a description / definition of each life form type.

*Table was consolidated and simplified as suggested by the reviewer*

- Fig. 1: What exactly is shown here? Are these only the associates of M. crassifolia as stated in lines 263/264, i.e., species co-occurring with the target species, or are these all the species that you found across the 78 plots?

*These are all species found in the 78 plots. We clarified this in the text. “*Fig 1 presents the life-form spectrum of all species found in the 78 plots. *”*

As you worked with percent cover per species, it would as well be interesting to illustrate how the percent cover of the target species does change with the percent cover of specific life form types.

*This is a good suggestion. We did not present a statistical analysis of the relation but we did present a descriptive analysis of the relation between target species cover and specific life form types in Table 4. This information was also explained in the results section and included in the Discussion section.*

- Table 6: What does grey vs. no shade depict? What does “0” stand for?

*The whole table was simplified to clarify the findings. Shading was removed dashes were added to indicate the absence of quadrats and 0 was used to indicate no presence of target species.*

- Lines 339/340: Rather “lowest representation” than “highest representation”? Otherwise, I do not understand how this is related to quadrat groups that excluded the target species.

*We thank the Reviewer for identifying this mistake. Indeed, we meant lower representation.*

- Table 8: Once, there is “chamaeophytes” written instead of “chamaephytes”. Please, correct.

*Chamaephytes suggested throughout the text as pointed out by Reviewer.*

Discussion:
- Parallel to the Introduction, the Discussion would benefit from better linking the study’s results to recent urban biodiversity research, especially to our knowledge about species conservation in urban areas, to the concept of novel ecosystems, and to functional traits and functional groups.

*As suggested by the Reviewer, the Discussion section was revised to better link the study’s results to recent urban biodiversity research unlike the introduction section, in the discussion section we reduced the number of references used and relied on the most relevant instead.*

Lines 365/366: “Such studies, however, are mostly conducted in natural habitats, and in many instances, deliberately exclude disturbed areas from sampling” – You do not compare natural to urban habitats, so why discuss this?

*We agree with the reviewer and deleted this sentence.*

- Line 374: “aspect of urban vegetation that challenges field data analysis”: It might challenge classification but no analysis as such (see above). – same in line 384

*The sentence was revised as per Reviewer suggestion*

- Line 387: How to develop an understanding of urban habitat analogs when there’s no description of natural habitats the target species usually occurs in (see above)?

*As per suggestion of Reviewer, we added a description of the natural habitat of the target species*

*“The natural habitat of the target species is described as coastal area rocks [46]. However the species behaves as a ruderal and was found in a diversity of habitats ranging from highly managed street medians to semi-natural coastal cliffs and including commercially introduced species and native ones.”*

- Lines 410-414: How to relate your method to LIDAR-data as long as herbaceous species cannot be distinguished by remote sensing (such as LIDAR)?

*A clarification was included in the discussion section to explain how LIDAR-data would allow a first screening to exclude areas that are known not to be favorable, such as canopy cover:*

*“In the last decade, several studies have sought to explore the potential of light detection and ranging (LiDAR) to inform landscape biodiversity assessments. In fact, the utilization of this technology has developed from quantification of gaps (above bare ground, low vegetation and medium vegetation), canopy cover and its vertical density in open landscapes (Miura, 2010) to mapping tree cover and vegetation spatial and vertical structure in cities and estimating above ground biomass despite particular challenges posed by urban areas [94, Wilkes et al 2018]. Furthermore, accurate mapping of vegetation communities within highly disturbed urban landscapes was recently achieved through incorporating a hierarchical object-based image analysis (OBIA) approach with high-spatial resolution imagery and canopy height surfaces derived from LiDAR data (Snavely et al, 2019). Provided the range of outputs these recent methods are producing, LiDAR technology allows a first screening to exclude areas that are known not to be favorable to the target species, for example canopy cover in the case of M. crassifolia. This will help in rapid identification of potential locations for habitat analogues in cities and guidance the management of urban vegetation for biodiversity conservation.”*

- Lines 422/423 “additional habitats”: You identified habitats where the target species occurs, habitats where it does not occur, and changes in percent cover of the target species across habitats. How did you identify additional habitats? Please explain in the Methods chapter.

*We added in the Methods a statement explaining how we performed a conceptual extrapolation of our findings to identify habitat analogues by identifying landscape typologies that host vegetation assemblages similar to quadrat groups in which the target species is highly represented.*

*We also added in the discussion a paragraph emphasizing this extrapolation.*

*“Streetscapes, such as street medians, street tree planters, that lack both ground cover and canopy species, ubiquitous throughout the city, provide patches of optimal vegetation composition that could potentially accommodate the target species. A change in landscape management strategy, however, needs to preceed introduction efforts. These additional habitat analogues experiences regular weeding by poorly trained municipality workers especially by the end of spring. In similar locations in which the species persist, municipality workers spare the vegetative rosettes and flowering adults, but immediately uproots plants in postbloom.”*

- Lines 443: Add “of” between “case” and “established”.

*Change made as suggested by Reviewer*

- Lines 442-447: How would you manage a green space where several species of conservation interest are competing with each other? Would you selectively remove some of them?

*In the case where more than one species is a conservation target, then a conservation strategy is developed that will accommodate for equal opportunity for conservation competing species by allocating habitat analogues to each. This information was included in the text.*

*“In the case where more than one species is a conservation target, then a conservation strategy is developed that will accommodate for equal opportunity for conservation competing species by allocating habitat analogues to each. We did not encounter such a case in our study.”*

 6. PLOS authors have the option to publish the peer review history of their article ([what does this mean?](https://eur03.safelinks.protection.outlook.com/?url=https%3A%2F%2Fjournals.plos.org%2Fplosone%2Fs%2Feditorial-and-peer-review-process%23loc-peer-review-history&data=02%7C01%7Cntsalma%40aub.edu.lb%7C139149d2a2a74c60613908d79b5f17a1%7Cc7ba5b1a41b643e9a1206ff654ada137%7C1%7C1%7C637148705038046160&sdata=FkU%2FWjxb3l5BeKksVmSNHaSkYK8rtb0QhS%2FsaTnIP4M%3D&reserved=0)). If published, this will include your full peer review and any attached files.


If you choose “no”, your identity will remain anonymous but your review may still be made public.


**Do you want your identity to be public for this peer review?** For information about this choice, including consent withdrawal, please see our [Privacy Policy](https://eur03.safelinks.protection.outlook.com/?url=https%3A%2F%2Fwww.plos.org%2Fprivacy-policy&data=02%7C01%7Cntsalma%40aub.edu.lb%7C139149d2a2a74c60613908d79b5f17a1%7Cc7ba5b1a41b643e9a1206ff654ada137%7C1%7C1%7C637148705038046160&sdata=AuKvBTv4T2w%2BHMFaUO9eBs6FE4xSODns%2FuMgnuX%2F4FU%3D&reserved=0).

Reviewer #1: Yes: Zerihun Woldu

Reviewer #2: No

Reviewer #3: No

[NOTE: If reviewer comments were submitted as an attachment file, they will be attached to this email and accessible via the submission site. Please log into your account, locate the manuscript record, and check for the action link "View Attachments". If this link does not appear, there are no attachment files to be viewed.]


While revising your submission, please upload your figure files to the Preflight Analysis and Conversion Engine (PACE) digital diagnostic tool, [https://pacev2.apexcovantage.com/](https://eur03.safelinks.protection.outlook.com/?url=https%3A%2F%2Fpacev2.apexcovantage.com%2F&data=02%7C01%7Cntsalma%40aub.edu.lb%7C139149d2a2a74c60613908d79b5f17a1%7Cc7ba5b1a41b643e9a1206ff654ada137%7C1%7C1%7C637148705038056158&sdata=obN4o9eK02NRQ%2FC1EhhyEHf71%2FURHiEZFmPMjeqexxE%3D&reserved=0). PACE helps ensure that figures meet PLOS requirements. To use PACE, you must first register as a user. Registration is free. Then, login and navigate to the UPLOAD tab, where you will find detailed instructions on how to use the tool. If you encounter any issues or have any questions when using PACE, please email us at [figures@plos.org](mailto:figures@plos.org). Please note that Supporting Information files do not need this step.

*In compliance with data protection regulations, you may request that we remove your personal registration details at any time.* [*(Remove my information/details)*](https://eur03.safelinks.protection.outlook.com/?url=https%3A%2F%2Fwww.editorialmanager.com%2Fpone%2Flogin.asp%3Fa%3Dr&data=02%7C01%7Cntsalma%40aub.edu.lb%7C139149d2a2a74c60613908d79b5f17a1%7Cc7ba5b1a41b643e9a1206ff654ada137%7C1%7C1%7C637148705038056158&sdata=ZUcd7Pf5PwVS4YkqyPq2EPb%2F6J8GP2z1tridZcVNE3A%3D&reserved=0)*. Please contact the publication office if you have any questions.*
