# Supplementary material for: Biodiversity conservation in cities: Defining habitat analogues for plant species of conservation interest
Source: PLoS One. 2020 Jun 9;15(6):e0220355. doi: 10.1371/journal.pone.0220355 (PMC7282666; doi:10.1371/journal.pone.0220355)
Supplement: Attachment — Submitted filename: RESPONSE TO REVIEWERS MAY 1 2020.docx [file pone.0220355.s008.docx]

PONE-D-19-19705
Biodiversity conservation in cities: Defining habitat analogs for plant species of conservation interest
PLOS ONE
RESPONSE TO REVIEWERS – SECOND FEEDBACK

Reviewer #2: The authors have made a good effort in addressing the points I mentioned in the last review and I see that the manuscript has considerably improved. I have still a few suggestions that I think might be interesting to consider in order to further improve the manuscript.

ABSTRACT

There have been numerous and interesting improvements in the manuscript in the last review, but the abstract has barely changed. In particular, I think it might be interesting to include a short sentence about the new information provided on the local habitat conditions of M. crassifolia (i.e. the it occurs throughout a wide range of potential urban habitats).

- *As suggested by the reviewer we made major revisions to the abstract to reflect the revised version of the manuscript and to mention the new information on local habitat conditions of M. crassifolia*

INTRODUCTION

In the introduction, the authors have now made good use of the current literature in urban ecology. This section has now improved considerably.

- *We thank the reviewer for the positive feedback*

METHODS

The methods are more concise and clear now. I also find the new figures on Beirut’s historical development and particularly on the location of the sites very informative.

- *We thank the reviewer for the positive feedback*

RESULTS

Regarding the results section, I would move Table 1 to the Appendix, as I think it might be too long and the authors have already made a good summary of it in lines 340-343.

- *We moved Table 1 to the Appendix as suggested by the reviewer*

Please check if all the figures are properly numbered when uploading, as some of the attached figures didn’t match the numbering in the text.

- *We revised all figures and tables and made sure that their numbering is correct*

I am happy to see that table 4 has information about the local habitat conditions of the sites.

- *We thank the reviewer for his/her favorable comment*

DISCUSSION

The discussion has substantially improved as well. The authors now added clarifications regarding the important local habitat conditions of M. crassifolia and about the reasons why the plant might be missing in some sites.

- *We thank the reviewer for his/her favorable comment*

GENERAL REMARKS

I think the manuscript has now improved considerably and can be suitable for publication after the aforementioned improvements.

Reviewer #3: Dear Itani et al.

Thank you for considering my previous comments. I think that the manuscript has greatly improved. Still, some minor issues remain. Please, allow me to point these out:

- Abstract, line 45: add “of” between “design” and “urban habitat analogues”

- *We added “of” between “design” and “urban habitat analogues” as suggested by reviewer*

- Lines 72 to 74: This sentence is misleading. It sounds as if novel ecosystems do include remnants of natural vegetation. Please, have a look at Kowarik & von der Lippe (DOI: 10.1111/1365-2664.13144) for definitions of natural remnants vs. novel ecosystems and do rephrase the sentence.

- *The reference Kowarik and von der Lippe was consulted as suggested by reviewers and the sentence was revised for more clarification.  “Novel ecosystems include urban green spaces that emerge mostly after built structures have replaced previously existing ecosystems. As such they include non native vegetation assemblages, consisting of native, spontaneous, naturalized, and invasive species [4]. ”*

- Lines 74/75: Isn’t management something intentional? So why “unintentionally managed”?

- *Sentence revised “Urban green spaces are sometimes abandoned after human disturbances or continue to experience disturbance regimes and consequently contain a range of both early- and late-succession vegetation. ”*

- Lines 76/77: "When unmanaged, urban green spaces … can potentially contribute to urban biodiversity conservation" – does that mean, on the contrary, that when managed, urban green spaces cannot contribute to urban biodiversity conservation? It sounds like this but I do not think that this is what you wanted to say, especially as in the end of your manuscript you suggest the intentional design and management of habitat analogues for the conservation of the target species. Please, rephrase lines 76/77.

- *Sentence in line 76/77 was rephrased to clarify that both unmanaged and managed green space can potentially contribute to urban biodiversity conservation. “Both unmanaged and managed green space can potentially contribute to urban biodiversity conservation.” added as introductory sentence.*

- Please note that habitat = biotope (the former is in English and the latter is derived from German, but basically, both mean the same). So, I suggest you only use the term habitat, e.g. from lines 92 on: “Many plant species can be found more or less regularly in various urban habitats. For example, classification of urban habitat types inside the city of Berlin has revealed 19 habitats particularly worthy of protection and these were nominated as legally protected [22]. However, the classification of habitat types inside cities requires standard habitat classification systems which have not been developed in all countries, at least not in Lebanon [23]. Furthermore, the nomination of such habitat types becomes challenging when [...]”

- *Habitat was used instead of biotope and sentence was changed as per reviewer suggestion*

- In lines 141-149, also use the terms habitat and classification: lines 145 “vegetation classification” instead of “vegetation description”; line 148 “vegetation classification” instead of “biotope type mapping”; lines 148 and 149 “habitats” instead of “biotopes”. The way the text is written at the moment makes me feel that different terms are used for the same thing, that’s why I am suggesting these replacements.

- *Terms were revised as suggested by reviewer*

- Lines 159/160, I suggest you write “[…] other studies suggest that descriptions of functional types, such as life form, may permit […]”. At the moment, it reads as if functional types are an example of life form – but rather, life forms are an example of functional types.

- *Sentence was revised as suggested by reviewer*

- Line 163: I do not know what you mean with “other physiognomic characters”. Can you use another term or provide an example?

- *We used another term as suggested by the reviewer “... life-form, among other descriptions of functional types, were associated with plant responses to environmental change...”*

- Lines 197 to 203 are confusing. There, it seems that Khaldeh, Beirut (including Ras Beirut), Amchhit and Byblos are your study sites. However, in lines 215 ff, you write that Ras Beirut is your study site. Please, be more precise in lines 197 – 203.

- *Lines 197 to 203 were revised to clarify that the study site was in Beirut*

- References to Fig. 1, 2, and 3 in the text are mixed up. Fig. 1 in the text = Fig. 2 where the figure is shown; similarly Fig. 2 in the text = Fig 3 and Fig 3 in the text is Fig.1.

- *References to figures were adjusted*

- Line 263 “we placed quadrats” – a certain number? Similarly, lines 266/267 “we increased the number of quadrats” – up to a maximum number of …?

- *Numbers were provided as suggested by the reviewer “In vegetation patches with clearly visible boundaries, one to two 1 m × 1 m quadrats were placed [32].” “... we increased the number of quadrats, up to six, … ”*

- Lines 304/305, the site numbers “(Site 17)” / “(Site 16)” do not seem to occur anywhere else in the manuscript (at least, I could not find them). I think that it is not necessary to provide these numbers as long as they are not referred to anywhere else (e.g. tables or figures). Therefore, I suggest to delete the numbers.

- *As suggested by reviewer sites 16 / 17 were deleted*

- Lines 447: “is classification is” – delete one “is”

- *Change made extra “is” removed*

- Lines 488/489: Shwartz et al. warn against expanding cities and they suggest to improve the quality of urban green spaces. But they do not present these two points as opposing strategies. Therefore, I suggest you phrase the sentence like this: “Improving the quality of existing green spaces throughout the entire urban matrix has been suggested as an effective approach to enhancing biodiversity experience [122].”

- *Sentence was rephrased as suggested by reviewer*

- Lines 550-552: This (trash) comes surprisingly and in my opinion has no close relationship to the core topic of your paper. I would delete it.

- *Sentence  regarding trash deleted as suggested by reviewer*

- Line 556: “increasing species’ site area” – do you mean all species or do you mean specific species (e.g. rare species, endemic species, protected species)?

- *In this sentence we mean only the target species. The sentence was revised to clarify this.  “... , increasing a target species’ site area in a city is highly desired ...”*

- In Fig. 2 it is very hard to visually distinguish between dots for Status = Extinct and dots for Status = Recolonized. Please, consider changing colors so that differences among the colors become more obvious (or check in the proof if quality go better than it is in the pdf-file that I got for review).

- *Color scheme was changed to better appear after publication*

- References: Please, do check references carefully as there are several typos within then. For example, journal names should be written with capital first letters (e.g. not Journal of applied ecology but Journal of Applied Ecology). Moreover, reference number 10 says “Doctoral dissertation” and “Master thesis” – it cannot be both a t once, can it? For reference number 19, rather cite the journal paper (Knapp, S., Kühn, I., Wittig, R., Ozinga, W.A., Poschlod, P. & Klotz, S. (2008) Urbanization causes shifts in species' trait state frequencies. Preslia 80, 375-388) than the book chapter, as the journal paper might be accessible to more readers. Also, in reference number 29, it should be “analogues” not “analogueues”. And with reference number 116, there’s an x after 116. There might be more typos as I did not look at them in detail.

- *We checked and corrected all references.*
